# AI-Assisted Rapid Quality Analysis in Implementation Science: Methodological Study

**DOI:** 10.2196/81149

**Published:** 2026-04-06

**Authors:** Adeola Adegbemijo, Anna M Maw, Katy E Trinkley, Amoolya A Varghese, Stephanie Tulk Jesso

**Affiliations:** 1 Systems Science & Industrial Engineering Watson College Binghamton University Binghamton, NY United States; 2 Division of Hospital Medicine University of Colorado Anschutz Medical Campus Aurora, CO United States; 3 Department of Family Medicine University of Colorado Anschutz Medical Campus Aurora, CO United States

**Keywords:** health care, implementation science, artificial intelligence, small language model, natural language processing, qualitative analysis, interviews

## Abstract

**Background:**

Translating evidence-based therapies from “bench to bedside” remains challenging, and implementation science (IS) experts are crucial for this process. Qualitative analyses are essential, but require extensive time and cost for manual coding. Now, many turn to artificial intelligence (AI) to accelerate the pace of qualitative analysis, but significant questions remain about the quality, validity, and ethics of applying large language models like ChatGPT (OpenAI) to qualitative data. To this end, we have developed a method for AI-assisted rapid qualitative analysis that addresses these concerns.

**Objective:**

This study aimed to develop AI-assisted rapid qualitative analysis for implementation science as an open-source encoder-based small language model (SLM) to aid IS experts. We focus on 2 efficient and high-performing SLMs: distilled bidirectional encoder representations from transformers (DistilBERT) and efficiently learning an encoder that classifies token replacements accurately (ELECTRA). The objective is to assess these models’ accuracy in reproducing expert coding, their generalizability to new coding scenarios, and enhancing their accessibility for nontechnical experts through user-friendly tools.

**Methods:**

Two previously coded IS datasets were used to train DistilBERT and ELECTRA models. These datasets were coded by IS experts using a mixed deductive and inductive approach, with initial categories derived from the domains of an IS framework: Practical, Robust Implementation, and Sustainability Model. We fine-tuned and evaluated DistilBERT and ELECTRA on these datasets, measuring performance by area under the precision-recall curve and Cohen κ. To facilitate use by nonprogrammers, we then developed an open-source Python package (pytranscripts) to streamline transcript processing, model classification, and evaluation. Additionally, a companion Streamlit web application allows users to upload interview transcripts and obtain automated coding and analytics without any coding expertise.

**Results:**

Our findings demonstrate the success of leveraging SMLs to significantly accelerate qualitative analysis while maintaining high levels of accuracy and agreement with human annotators, although results are not universal and depend on how researchers approach qualitative coding. On the original dataset, DistilBERT achieved near-perfect agreement with human coders (Cohen κ=0.95), while ELECTRA showed substantial agreement (Cohen κ=0.71). However, both models’ performance declined on the second, more ambiguous dataset, with DistilBERT’s Cohen κ dropping to 0.48 and ELECTRA’s to 0.39. Two primary drivers of performance drop appear to be related to the number of codes applied to the dataset, and whether coders apply multiple codes to each piece of data or constrain themselves to applying one.

**Conclusions:**

This work demonstrates that SLMs can meaningfully assist qualitative researchers with coding tasks as long as attention is paid to how experts code data that will train the SLM. This can be especially valuable in settings where deploying large language models is impractical or undesirable.

## Introduction

### Background

The field of implementation science (IS) is relatively new. The goal is to speed up the pace of translating health evidence and innovation into real-world health care delivery to improve human health [1]. IS is gaining recognition in terms of its critical role in bridging the gap between research and practice and improving the quality of health care services [2]. While IS often focuses on improving the quality of individual health care interventions within specific health systems contexts, another major focus of IS research is to identify new approaches that can help even novice IS practitioners articulate and promote strategies to improve implementation success and sustainability [3].

Due to recent advances in artificial intelligence (AI) tools and their plentiful access to the public, IS experts are now looking for opportunities to use AI to increase the speed and quality of their translational efforts [4,5], while there is great interest in the use of large language models (LLMs) to improve the speed of qualitative analysis, studies are lacking that evaluate their performance. Evidence is especially limited on how coding‑scheme design (single vs multicoding) affects model agreement with experts. We address this gap by benchmarking distilled bidirectional encoder representations from transformers (DistilBERT) and efficiently learning an encoder that classifies token replacements accurately (ELECTRA) on Practical, Robust Implementation and Sustainability Model (PRISM)–mapped datasets, quantifying agreement with expert coders, and releasing open tools that operationalize a privacy‑preserving workflow.

IS researchers use a cadre of techniques to thoroughly investigate various interventions within highly diverse and dynamic contexts. Qualitative research methods are an essential component in identifying multilevel contextual factors that influence the implementation of a health intervention [6-8]. Given that much of this qualitative data takes the form of text produced from interview transcripts, one major opportunity is the integration with natural language processing (NLP) in qualitative analysis. NLP is gaining popularity in IS and health care research more broadly to analyze large volumes of text data [9]. While it can be a powerful tool for extracting useful insights from qualitative data, such as interview transcripts, clinical notes, and survey responses, researchers are in need of examples to study in addition to established guidelines to ensure the validity and reliability of results using AI tools for qualitative analysis.

To situate AI’s role in IS, we first summarize qualitative coding practice and its current limitations, then review AI and NLP approaches and the specific tradeoffs between small language models (SLMs) and LLMs.

### Related Work

#### AI, Machine Learning, and Qualitative Coding: Importance and Challenges

Qualitative coding is critical for making sense of unstructured textual data. It enables researchers to extract themes, concepts, and insights in a systematic way [10]. In fields such as psychosocial studies, for example, coding counseling conversation transcripts can reveal patterns of effective communication or patient struggles [11]. In health care, coding patient interviews or clinical notes can surface concerns and outcomes that quantitative measures might miss [12]. Educational researchers code student feedback or classroom dialogue to study learning experiences and social dynamics. Since coding translates rich narratives into analyzable data, it is often described as the bridge between qualitative raw data and meaningful research conclusions. Despite its importance, manual coding is challenging on multiple fronts. It is time-consuming – researchers must read through large volumes of text and make judgment calls on how to label each segment. Another issue is that traditional qualitative analysis software (like NVivo [Lumivero] or MAXQDA [VERBI Software]) provides excellent tools for organizing and retrieving coded text, but they offer minimal automation of coding itself; where automation is available, there is still a paucity of studies demonstrating its performance against the human gold standard. IS researchers have criticized that most qualitative coding tools are essentially used as “electronic filing cabinets” rather than intelligent assistants [13]. Human-computer interaction research has found that analysts would prefer an AI system that suggests code for a passage that the researcher has selected, rather than an autonomous system that codes an entire dataset unseen. This preference stems from a need to retain interpretative authority and trust in the process [14].

Qualitative coding is a cornerstone of research in social sciences, health care, and education. The desire for techniques to increase the speed and reduce the manual effort spent in conducting high-quality qualitative analysis is not new. Some techniques that have been discussed under the umbrella of “rapid qualitative analysis” (or “rapid qual”) include conducting interviews online [15], developing and reinventing deductive frameworks that simplify IS concepts, and adopting new techniques to streamline transcript generation [16,17]. Building on these established methods, researchers are now exploring potential ways to use AI to speed up and simplify qualitative work to empower IS researchers in their translation of new techniques and tools that improve health care.

Qualitative coding at its best is a collaborative and progressive system that includes checks and balances to represent subjective perspectives. Qualitative researchers want to know that this core is preserved when building for this purpose [18]. At present, individuals who are not familiar with coding or Machine learning (ML) and NLP techniques can turn to LLMs such as ChatGPT, although these are not without limitations or tradeoffs. Of great concern for qualitative researchers are baked-in biases, the policy of retaining user prompts and responses that may violate participant consent, and the tendency to produce strange and inaccurate responses known as “hallucinations” [19,20]. Researchers may also take issue with the negative environmental impacts and exploitative policies associated with current proprietary generative AI, such as ChatGPT [21-23]. Studies on the perceived impacts of AI solutions in health care across patients, health care professionals, and organizations show that despite the complexities of integrating these advanced tools in the sector, health systems leaders believe that the benefits are apparent [24]. This raises the question of how to optimize the use of AI for qualitative analysis, and beyond, given the current state of technology.

Early attempts at automating qualitative coding applied methods like rule-based systems or classical ML. Researchers have explored rules for coding forum messages, and others have tried basic supervised models on small coding tasks [25]. These approaches showed some promise, but they were limited in handling the complexity of natural language. Rules could not capture subtle context, and simpler classifiers required extensive feature engineering and still fell short of human-like understanding. The rise of deep learning and large pretrained language models brought new opportunities to this space. Models such as bidirectional encoder representations from transformers (BERT), introduced in 2018, enabled much more nuanced text understanding through pretraining on vast corpora [26]. By fine-tuning such models on a labeled dataset of text segments and codes, researchers could leverage their language comprehension for coding tasks. Indeed, recent studies have started to apply BERT-based models to qualitative coding with encouraging results. In 2020, some researchers created a coding scheme of over 50 categories for psychosocial online counseling conversations and manually annotated more than 10,000 text passages. They then fine-tuned state-of-the-art classifiers, including BERT, on this data to evaluate automatic coding performance against human coders. The best BERT model achieved a weighted *F*_1_-score of ~74%, outperforming a baseline support vector machine classifier (68% *F*_1_-score) on the test set, demonstrating that modern NLP models can indeed learn to replicate human coding to a useful degree. Notably, the automated approach could handle nuanced categories like different types of emotional support or counseling strategies, which would have been very difficult with earlier techniques [11].

Recent advances in NLP, particularly large pretrained language models, have opened new avenues for coding automation. Models like BERT and GPT-3 can understand textual context and perform classification tasks, suggesting they could categorize text segments into qualitative codes with reasonable accuracy [26]. However, most state-of-the-art language models are extremely large, with hundreds of millions or billions of parameters, making them resource-intensive to use. Many qualitative researchers operate in low-resource settings without access to powerful hardware or extensive computing budgets. Moreover, sensitive qualitative data (eg, clinical interviews) often cannot be freely uploaded to external AI services for analysis, necessitating local, efficient solutions. These factors have spurred interest in SLMs as a practical tool for qualitative coding. This review focuses on DistilBERT as an exemplar one, examining its benefits, performance trade-offs, and applications in qualitative research. We compare DistilBERT to other small models and larger models, discuss the challenges of automating qualitative coding, and highlight evidence from diverse domains that shows the potential of SLMs like DistilBERT for qualitative researchers.

#### SLMs vs LLMs

The societal impact and impressive performance of LLMs at present are undeniable. LLMs such as GPT-3, GPT-4, or BERT’s larger successors currently achieve top performance on many NLP benchmarks [27]. They benefit from massive scale, which often yields greater linguistic and world knowledge. In theory, an advanced LLM might produce highly accurate coding suggestions or even infer themes without training. However, in practice, deploying such models in qualitative research settings presents significant drawbacks. First, the computational cost is immense: running inference on a large model requires graphics processing units and substantial memory or reliance on cloud application programming interfaces. Many academic and non-profit researchers lack these resources, working “on the edge and/or under constrained computational budgets” [26]. As model sizes grow, the environmental and financial costs of using them also skyrocket, a valid concern about the sustainability of NLP research. Second, large proprietary models (eg, OpenAI’s GPT series) may not be usable with sensitive data due to privacy concerns, since they operate through external servers. Accessibility thus becomes a barrier, and the best technology may be out of reach for those without funding or willing to risk data confidentiality [28]. Even when these tools can be contracted for privacy and confidentiality, users must trust that large AI companies, sometimes having less-than-stellar reputations for ethics, will not violate agreements or expose their data in cybersecurity breaches. These findings highlight the need for building bespoke NLP systems designed for specific domains, such as qualitative analysis in health care, where ethical considerations, data privacy, and domain relevance are paramount. Others have shown that LLM hallucinations are inevitable due to the limitations of training data, model architecture, and inference mechanisms [29]. SLMs, on the other hand, are open-source, pretrained models that can be downloaded, fine-tuned, and deployed locally, providing a cost-effective and secure alternative to large proprietary models. These models are particularly attractive for privacy-sensitive applications, such as health care and qualitative research, as they can be run locally, avoiding data transmission to third parties. Custom-built NLP systems grounded in encoder models can overcome many LLM limitations by focusing on domain-specific data and targeted frameworks.

#### Purpose

**Figure 1:**
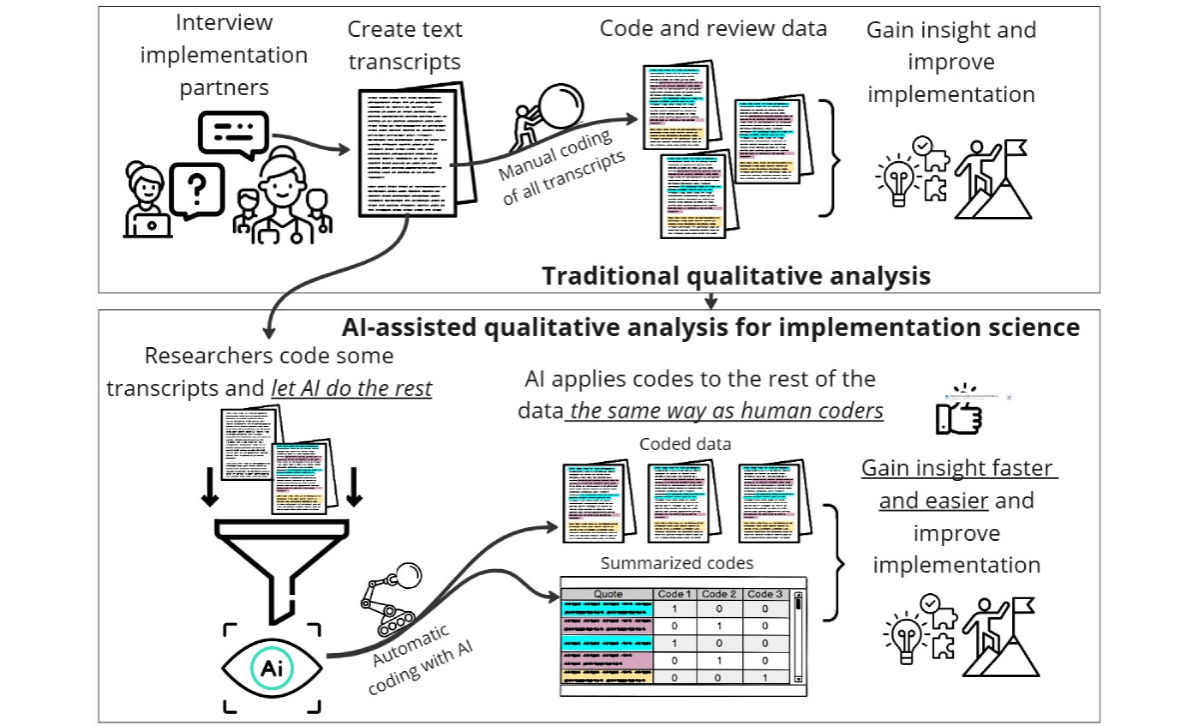
Conceptual framework for qualitative coding analysis using small language models. AI: artificial intelligence.

Given these constraints and the need for privacy-preserving, resource-efficient tools, we evaluate whether small, locally deployable encoder models can close the gap in AI-assisted qualitative coding for IS researchers. The purpose of this effort is to accelerate the process of translating evidence-based health care interventions into practice by addressing the time-consuming and costly nature of qualitative analysis. Figure 1 shows the broad conceptual model we began with that guided our method, comparing the workflow between traditional qualitative analysis and AI-assisted qualitative analysis within the context of IS. We wanted to apply methodologically sound NLP techniques in ways that made it easy for IS researchers to understand and find use within their work. In the following sections, we outline the specifics of the approach we developed for AI-assisted qualitative analysis.

## Methods

### Data Collection

For a year, our transdisciplinary team, consisting of academic engineers with specialties in human-centered design, human-computer interaction, and data science, and IS researchers, who are also practicing physicians and academics, collaborated to develop and outline an approach to rapid qualitative analysis using NLP and the contextual domains of one IS framework, PRISM. PRISM is commonly used to guide contextual assessments; it is a widely used IS framework that facilitates the identification of the determinants (ie, barriers and facilitators) of intervention implementation present in the multilevel context [30-32]. PRISM was used to guide the qualitative data analysis performed by collaborators; its multilevel approach makes this framework particularly suitable for complex health care interventions like adopting new diagnostic tools, such as lung ultrasound, and the management of patients with heart failure and reduced ejection fraction (HFrEF). Point-of-care ultrasound, particularly lung ultrasound (LUS), has emerged as a valuable diagnostic tool in hospital medicine. LUS offers several advantages over traditional imaging modalities, such as chest x-rays, including increased accuracy, expedited diagnosis, and reduced radiation exposure for patients. Professional societies now endorse LUS use in acute care settings to assess dyspnea and monitor volume status in patients with heart failure [7]. However, studies find that several challenges affect the integration of LUS into care practice, and this work is integral to helping break down these barriers as they work toward effective implementation. In the HFrEF research, authors also highlight the need for targeted interventions that improve clinician comfort with prescribing newer medications, enhance communication between generalists and specialists, and address the real-world application of the medication guidelines [33].

We engaged in deep conversations about the needs and desires of IS experts as well as the potential use and stumbling blocks presented by AI, and used previously coded qualitative datasets provided by our collaborators to train and validate the SLM models, addressing the need for a context-specific and sensitive AI tool to help accelerate the research process.

**Figure 2:**
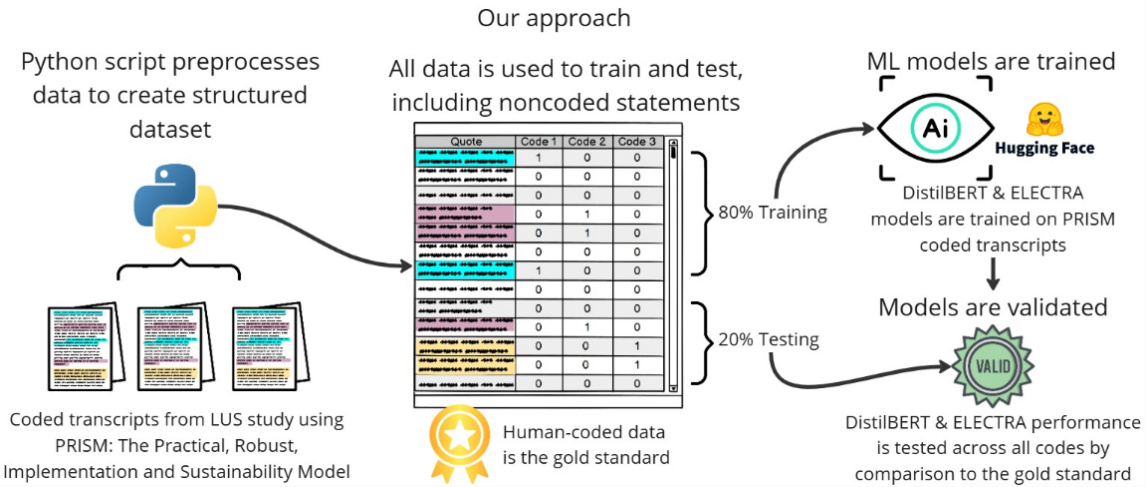
Process map for AI-assisted rapid qualitative analysis for implementation science development showing pipeline for training and validating small language models using human-coded Practical, Robust Implementation and Sustainability Model data. AI: artificial intelligence; DistilBERT: distilled bidirectional encoder representations from transformers; ELECTRA: efficiently learning an encoder that classifies token replacements accurately; LUS: lung ultrasound; ML: machine learning; PRISM: Practical, Robust Implementation and Sustainability Model.

A process map detailing our approach can be seen in Figure 2 above, where we show the development pipeline, detailing the data preparation, model training, and evaluation process used with AI-assisted rapid qualitative analysis for implementation science (AI-RQA; outlined in further detail throughout this section). By incorporating these detailed steps into our algorithm, we aimed to create a robust and reproducible framework for analyzing large-scale textual data. It is a process map that illustrates a reproducible and scalable strategy for developing ML models that support high-fidelity, AI-augmented qualitative analysis. The resulting classification serves as a foundation for subsequent analyses investigating diverse sets of challenges in health care intervention wrapped within the PRISM framework.

### Data Processing

The dataset used for this case study consists of 75 interview transcripts of clinicians, 42 of which were focused on contextual determinants of LUS adoption in diverse health care settings, while 33 of these were on identifying and understanding clinician-perceived challenges in prescribing HFrEF medications. These datasets, originally collected for different studies, were shared with approval from the institutional review board.

A mixed deductive and inductive coding process was used to integrate the contextual domains of the PRISM framework, which includes both external and internal environments. Subdomains within these categories were derived from known facilitators and barriers across the following high-level categories, seen in Table 1.

The training data came from a spreadsheet with over 1461 annotated text samples that were tagged into 42 subcategories, included in Multimedia Appendix 1. To ensure the consistency and accuracy of the dataset, an automated data-cleaning process was implemented. This process standardized the text, facilitating uniformity across all documents. Preprocessing involved converting Microsoft Word files into a structured format, ensuring proper formatting, and extracting pairs of interviewer and interviewee dialogues. The interviews, which involved health care professionals, were systematically processed using a Python (Python Software Foundation) script to extract both interviewer questions and interviewee responses from the documents.

The interview data were parsed into smaller text units (sentences and phrases) and mapped onto predefined coding categories listed above. These codes align with the categories identified in the qualitative analysis of the data previously tagged by human coders, and each text unit was cross-checked against human-annotated labels to ensure high-quality tagging. For the final dataset, we introduced all question-answer pairs, including those that do not appear in the annotated labels, resulting in 5173 rows.

**Table 1:** Mapping of Practical, Robust Implementation and Sustainability Model domains to lower-level codes for model training.

PRISM^a^ classification	Lower level classification
Multilevel organization characteristics	Provider characteristics, health systems characteristics, comanagement, comanagement_clinician responsibility, comanagement_communication
Multilevel organization perspectives or values	Imaging modalities in general, value equation, clinical use, and efficiency, or provider perspective, patient or physician interaction in LUS^b^, workflow-related problems
Implementation and sustainability infrastructure	Training, credentialing, or quality assurance infrastructure, financial impact, workflow, provider education, provider_education format or accessibility, EHR^c^, EHR_CDS^d^, EHR_CDS_Format or timing, EHR_CDS_likes or dislikes, EHR_likes or dislikes, patient digital tools, patient_education or communication, time constraints
Clinician perspective	HFrEF^e^ comfort managing, med management, med management_titration or dosing, medmanagement_monitoring or follow ups, med management_polypharmacy, informational needs, info needs_risk/benefit data
External environment	Guidelines, guidelines_familiarity or awareness, guidelines_use, algorithm, cost, COVID
Patient perspectives	Patient_priorities or goals, patient_symptoms or side effects, patient_provider relationship or rapport,
Patient characteristics	Patient_social characteristics, patients_clinical characteristics, competing priorities

^a^PRISM: Practical, Robust Implementation and Sustainability Model.

^b^LUS: lung ultrasound.

^c^EHR: electronic health record.

^d^CDS: clinical decision support.

^e^HFrEF: heart failure and reduced ejection fraction.

### Model Selection and Training Setup

Given our focus on privacy-preserving, resource-efficient AI for qualitative coding, we selected 2 encoder-based SLMs, DistilBERT and ELECTRA [26,34]. Both models are substantially smaller and faster than the larger language models while retaining strong performance and efficiency in handling sentence-level classification and context-based tasks, making them suitable for deployment on modest hardware and within institutional data governance constraints.

DistilBERT represents a distilled variant of BERT, reducing parameter count and inference cost while preserving much of BERT’s contextual representation quality. ELECTRA uses a different pretraining objective based on detecting token replacements rather than reconstructing masked tokens, which makes it sample sufficient and effective for classification tasks. Together, the models allow us to compare 2 complementary SLM architectures that are feasible for on-device use and well-suited for assigning PRISM-based codes to short text segments.

We fine-tuned the DistilBERT and ELECTRA models for multiclass thematic classification across 6 higher-level categories and 42 lower-level categories. Following established transformer training practices [35], we tuned key hyperparameters within predefined ranges, including batch size, number of epochs, weight decay, learning rate, and maximum sequence length, using an evaluation strategy and best model saving to select the best configuration for each model (Table 2).

Upon completion of hyperparameter selection, we trained each model on a stratified 80/20 train-test split to ensure a balanced representation of all categories within the training and testing sets. Specifically, we randomly sampled 80% of the documents belonging to each category for training purposes and reserved the remaining 20% for testing. This procedure ensured both models maintained consistent proportions of each category between the various subsets, thereby reducing potential biases associated with imbalanced class distributions.

**Table 2:** Hyperparameter optimization settings.

Models			Lower range	Upper range		
**DistilBERT^a^**						
	Learning rate		1 X 10^–5^		1 X 10^–4^	
	Epoch		10		20	
	Max length		256		512	
**ELECTRA^b^**						
	Learning rate	2 X 10^–5^				2 X 10^–4^
	Epoch	10				20
	Max length	256				512

^a^DistilBERT: distilled bidirectional encoder representations from transformers.

^b^ELECTRA: efficiently learning an encoder that classifies token replacements accurately.

### Evaluation Metrics

To provide an in-depth assessment of the model performance beyond overall accuracy, we evaluated the macro, micro, and weighted *F*_1_-score, along with per-class precision and recall, to summarize performance under the substantial class imbalances present in qualitative coding datasets.

We further computed the area under the precision-recall curve (AUPR) for each PRISM category and Cohen κ to quantify the agreement between each model and the consensus human “gold standard” across both datasets. Together, the metrics are capable of capturing both class-specific and overall model-human agreements, rather than relying on the overall accuracy value.

### Operationalization and Tooling

To bridge the gap between research and practical application, we developed 2 complementary open-source tools. First, we used pytranscripts, an open-source Python library for automated classification and analysis of qualitative health care research transcripts [36]. This library leverages pretrained DistilBERT and ELECTRA to perform text classification, handling tasks end-to-end from raw data extraction through to label assignment. The library handles input formats for documents commonly used in qualitative research. Internally, pytranscript applies the data processing steps described in data processing (segment parsing, code alignments, and cleaning). It also integrates robust evaluation procedures, for example, pytranscripts computes agreement metrics such as Cohen κ to compare model predictions with human annotation, ensuring that the automated classifications align well with expert-coded data.

We also built a user-friendly web interface using Streamlit (Snowflakes) to make these SLM models accessible to nontechnical domain experts. This app [37] provides an interactive platform where users can upload their own transcript datasets, choose between the available pretrained models, and adjust confidence thresholds for automated coding. Under the hood, the Streamlit interface uses the pytranscripts pipeline to process data and visualize classification results. While our current experiments focus on text prepared from interview recordings, the tooling is designed to integrate upstream with automatic speech recognition systems in future work. The system is designed to support local or institution-hosted deployment, helping to keep sensitive qualitative data within the organization’s infrastructure (Multimedia Appendix 2).

The pytranscripts library [36] is released as an open-source, community-extensible tool on GitHub. By making our code and models publicly available, we aim to foster reproducibility and collaboration in qualitative analysis workflows. Researchers and developers are encouraged to engage with these resources, whether by applying the tools to their datasets or by contributing improvements and new features via GitHub. This operationalization of SLMs not only demonstrates the feasibility of deploying lightweight NLP models in health care research contexts but also invites the community to collectively refine and advance these tools in future studies.

### Ethical Considerations

This study was determined to be exempt from institutional review board approval by both participating institutions (University of Colorado Anschutz; SUNY Binghamton) because it used only deidentified secondary data for model training. All data used contained no personally identifiable information (PII). Details regarding the original data collection and thematic analysis can be found in [7,33].

## Results

### Overview

In this section, we present the findings from evaluating DistilBERT and ELECTRA for qualitative analysis of health care research data. Our study provides evidence supporting the feasibility of implementing SLMs in qualitative coding tasks, with models demonstrating an ability to mirror human annotators at high accuracy levels, and showing scenarios where performance falters.

### Model Performance Across Datasets Based on Evaluation Metrics

The overall model performance was first evaluated using accuracy, weighted precision, recall, and *F*_1_-scores for each dataset-model pair (Table 3). On the LUS dataset, DistilBERT achieved high performance with an accuracy of 97% and an *F*_1_-score of 96%, indicating close correlation with human coding. ELECTRA also performed well on LUS, although at a lower level, with an accuracy of 85% and an *F*_1_-score of 80%. On the HFrEF dataset, which included more PRISM categories and a more complex coding scheme, performance reduced for both models, with DistilBERT at 48% accuracy and an *F*_1_-score of 46%, while ELECTRA achieved 38% accuracy and an *F*_1_-score of 33%, indicating the difficulty of the models to perform under this setting.

**Figure 3:**
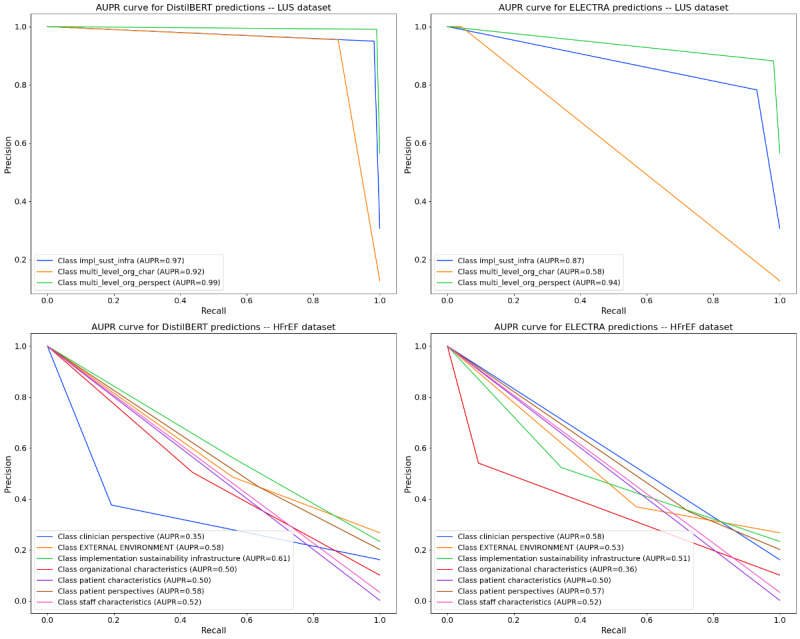
Precision-recall curves and area under precision-recall values for DistilBERT (labeled as “BERT” in plots) vs ELECTRA on the lung ultrasound dataset (top row) and heart failure and reduced ejection fraction dataset (bottom row). Each colored line corresponds to a different category, with its area under precision-recall indicated in the legend. AUPR: area under precision-recall; DistilBERT: distilled bidirectional encoder representations from transformers; ELECTRA: efficiently learning an encoder that classifies token replacements accurately; HFrEF: heart failure and reduced ejection fraction; LUS: lung ultrasound.

To examine the performance at the level of individual PRISM categories, we analyze the precision-recall curves and the corresponding AUPR for each model and dataset (Figure 3). On LUS, DistilBERT achieved a consistently high AUPR across all 3 categories, ranging approximately from 0.92 to 0.99. In contrast, ELECTRA showed more variability, between 0.58 and 0.94, with one category noticeably harder than the others. On the HFrEF, which introduces 7 upper-level categories, both models’ performances drop substantially (Figure 3, bottom). DistilBERT’s AUPR now spans 0.35-0.61 across the new classes, while ELECTRA’s AUPR ranges 0.36-0.58, indicating that the task became far more challenging. Some categories in the HFrEF dataset are relatively easier for DistilBERT, implementation and sustainability infrastructure is best predicted (AUPR≈0.61), and external environment and patient perspectives are also moderate (AUPR≈0.58 each). DistilBERT finds the clinician perspective the hardest (AUPR≈0.35), revealing a significant drop in precision-recall performance for that class. ELECTRA’s strongest new class is clinician perspective (AUPR≈0.58), ironically, the class DistilBERT struggled with, and also patient perspectives (AUPR≈0.57). ELECTRA’s weakest category is organizational characteristics (AUPR≈0.36).

Overall, Table 3 and Figure 3 show that both models strongly perform in the simpler LUS setting and face greater challenges in the more complex HFrEF context. The metrics consistently show that DistilBERT is the stronger model across datasets (for details, refer to Multimedia Appendix 1 for the normalized confusion matrix across both datasets), while ELECTRA remains competitive but more sensitive to category-specific difficulty and coding complexity.

**Table 3:** Overall classification performance metrics on lung ultrasound, heart failure, and reduced ejection fraction.

Dataset	Model	Accuracy	Weighted precision	Weighted recall	Weighted *F*_1_-score
LUS^a^	DistilBERT^b^	0.97	0.97	0.95	0.96
LUS	ELECTRA^c^	0.85	0.87	0.85	0.80
HFrEF^d^	DistilBERT	0.48	0.46	0.48	0.46
HFrEF	ELECTRA	0.38	0.35	0.39	0.33

^a^LUS: lung ultrasound.

^b^DistilBERT: distilled bidirectional encoder representations from transformers.

^c^ELECTRA: efficiently learning an encoder that classifies token replacements accurately.

^d^HFrEF: heart failure and reduced ejection fraction.

### Agreements With Human Coders

To validate the model outputs, we compared each model’s predicted qualitative codes against the human “gold standard” on 2 datasets (LUS and HFrEF). The gold-standard labels were created by consensus of multiple expert annotators, meaning several experts discussed and agreed on the code for each instance. This consensus-based approach provides a high-quality reference for evaluation, as it reduces individual bias and ensures the ground truth reflects collective expert judgment. We used the Cohen κ score to quantify agreement between each SLM compared to human coders.

The value of Cohen κ ranges from –1 to 1, where 1 indicates perfect agreement between model and humans, 0 indicates no agreement beyond what would be expected by random guessing, and negative values indicate active disagreement. In simple terms, a higher Cohen κ means the model is doing a better job at predicting the same codes that experts would assign. Using this framework, we evaluated DistilBERT and the ELECTRA model performance on both datasets. The observed Cohen κ scores are given in Table 4.

On the LUS dataset, DistilBERT achieved near-perfect agreement with the expert codes with a 0.95 Cohen κ score, effectively mirroring human coding decisions. ELECTRA also performed well with 0.71 Cohen κ, demonstrating substantial agreement. Agreement was lower on the HFrEF dataset, with DistilBERT having a Cohen κ of 0.48, which is a moderate agreement level, and ELECTRA with a fair Cohen κ of 0.39. This decline can largely be attributed to how the HFrEF dataset was labelled, specifically the multicoding approach used by researchers. This created ambiguity in label assignment, making it difficult for both models to match the human gold standard. Even human annotators faced challenges with this issue, which naturally lowered agreement.

Our AUPR curves highlight these discrepancies; they double as an error analysis focusing on segments where model predictions diverged from human codes. This review revealed distinct patterns of misclassification within the HFrEF dataset, particularly in semantically overlapping domains. For instance, DistilBERT frequently misclassified statements labelled as “Clinician Perspective” as “Implementation Sustainability Infrastructure” or “Patient Perspective.” Qualitative review suggests the model often focuses on topic-specific keywords rather than the speaker’s perspective, complicating the distinction between internal clinical views and external factors in a multicode framework. These findings underscore how coding-scheme design and label ambiguity can constrain the level of agreement achievable by even well-performing SLMs.

**Table 4:** Agreement scores.

Dataset and model			Cohen κ		Agreement level		SE		95% CI
LUS^a^									
	DistilBERT^b^	0.95		Almost perfect agreement		0.0208		0.9056-0.9905	
	ELECTRA^c^	0.71		Substantial agreement		0.0442		0.6193-0.7950	
HFrEF^d^									
	DistilBERT	0.48		Moderate agreement at best		0.0077		0.4690-0.4991	
	ELECTRA	0.39		Fair agreement at best		0.0075		0.3730-0.4023	

^a^LUS: lung ultrasound.

^b^DistilBERT: distilled bidirectional encoder representations from transformers.

^c^ELECTRA: efficiently learning an encoder that classifies token replacements accurately.

^d^HFrEF: heart failure and reduced ejection fraction.

### Tool Availability and Example Interface

To facilitate use by nontechnical IS researchers, we packaged the final workflow into the pytranscripts library and web-based Streamlit application. The library automates key steps, including transcript ingestion, preprocessing, model inferencing, and metric computation, so that teams can reproduce our pipeline in scripted analyses without rewriting infrastructure code.

**Figure 4:**
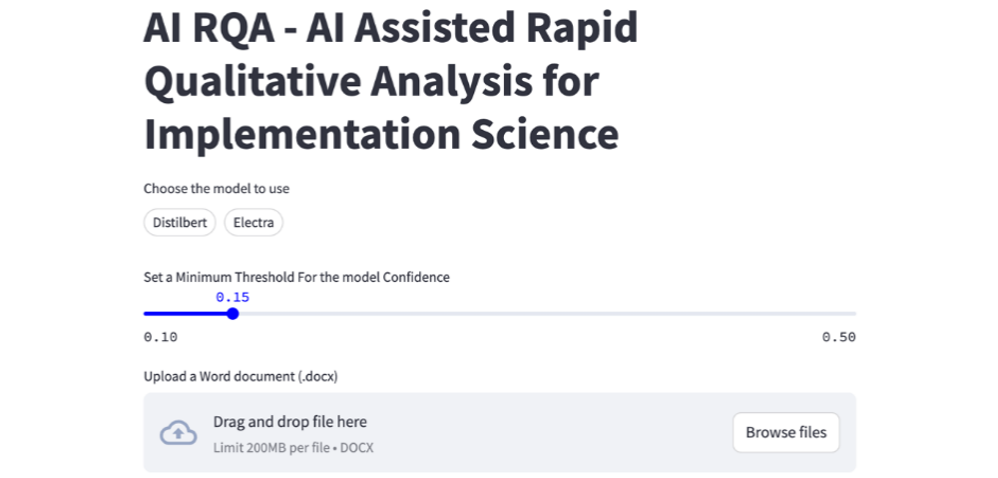
Snapshot artificial intelligence–assisted rapid qualitative analysis for implementation science Streamlit interface. AI-RQA: artificial intelligence–assisted rapid qualitative analysis for implementation science.

The Streamlit Interface (Figure 4) is designed for users who prefer a more interactive workflow. Users first upload one or more transcripts in common formats (eg, DOCX, TXT, etc). The user chooses the pretrained model (DistilBERT or ELECTRA) and also tunes parameters such as the confidence threshold for automated coding. After processing, the app displays the PRISM-mapped codes for each segment alongside the model confidence scores, making it easy for researchers to review, accept, or revise predicted labels. Together, these tools not only support rapid exploration of determinant patterns without requiring programming expertise, but also lower the barrier for implementation of AI-assisted qualitative analysis in IS settings.

## Discussion

### Principal Findings

AI-assisted qualitative analysis is not only a desirable alternative to fully manual analysis, but our study using SLMs shows that it is possible to achieve high-quality results that can overcome limitations associated with current LLM approaches. However, strict framework alignment is needed when coding training datasets to ensure validity. Our experience in developing an automated coding approach using NLP techniques for qualitative analysis revealed several key lessons worthy of note for those intending to adopt a similar strategy. Outside of sharing our findings and adding to the body of literature, our goal is the replicability of this process. We have tried to be extremely explicit and transparent in describing the methodology so that others understand the steps involved. As we work toward improving these techniques, our eventual goal is to enable lay users to confidently adopt these methods in diverse settings.

DistilBERT and ELECTRA were able to replicate human coding decisions with high accuracy, especially when dealing with well-defined datasets. In the context of qualitative research, SLMs are more practical for privacy-sensitive data as they can be deployed locally, avoiding the high computational costs and security concerns associated with cloud-based LLMs. Both models showed strong performance in replicating expert annotations, with DistilBERT outperforming ELECTRA in consistency for the LUS dataset. However, when tested on the HFrEF dataset, both models saw a sharp decline in performance. This suggests that the models struggled to generalize effectively to the new data, which introduced more complexity and ambiguity in the form of multicoding, which is a valid and common practice in qualitative analysis. This finding highlights the importance of establishing coding standards that will be compatible with AI (if the eventual goal is to use AI-assisted coding), and the difficulty of maintaining model accuracy when the coding scheme is more ambiguous.

While our AI-RQA approach is desirable to speed up qualitative analyses, NLP or AI developers must pay careful attention to ensure the validity and reliability of results [38]. The AI-assisted rapid qualitative analysis must provide the depth of understanding that is central to qualitative analysis and stay true to the complexities and nuance of IS qualitative data, while also creating a straightforward pathway to enable other IS researchers to apply this method and trust their results [35,36]. The true standout effort needed for this process is the meticulous data structuring and preparation needed to convert data sheets that are coded and interpretable by humans into something that can be used to train a model.

In terms of evaluating a model's performance, it is crucial to look at it in diverse ways to get a complete picture. Comparing model predictions on individual codes is more meaningful because it maps the nuance of variability between human and AI perceptions back onto the real characteristics that are meaningful to the researchers. In addition, models themselves perform differently, and knowing that one approach excels with one code but performs poorly with another allows researchers to make more informed decisions on how to use the output.

Our results reflect 2 health care IS contexts and coding schemes; broader validation will require additional domains and deliberate treatment of multicoding. Future work should (1) standardize AI-compatible coding practices and exemplars, (2) assess end-to-end pipelines, and (3) conduct usability studies to understand how researchers and clinicians integrate AI-RQA outputs into their analytic and decision-making workflows.

### Limitations

These findings are built on a limited study, although the scope is currently being expanded to include other IS evaluations of contextual determinants. It is important to state some of the limitations that might occur based on this. First, our modest dataset size constrains the broad applicability of our research findings. The scope also raises concerns regarding deriving insights exclusively from a few case studies; we risk loading the algorithm with patterns unique to this distinct investigation, and that raises questions about its performance in alternative medical and clinical settings. These limitations do not diminish the value of our study; they form the basis of our continued work to innovate qualitative analysis processes in IS.

### Conclusions and Future Work

In this paper, we applied 2 SLMs, DistilBERT and ELECTRA, to PRISM-guided IS interview transcripts to support AI-assisted rapid qualitative analysis. By fine-tuning these SLMs on 2 IS datasets (LUS and HFrEF), we showed that models deployed locally can approximate expert coding while preserving data confidentiality and remaining computationally efficient. We also operationalized the workflow in the open-source pytranscripts library and a Streamlit web-based application. This provides an interactive interface for IS researchers to implement SLMs into the qualitative analysis workflow without prior technical expertise.

A key drawback of this study is the sensitivity of model performance to the initial coding scheme and dataset scope. In the simpler LUS setting, strong agreements with human coders were achieved; however, in the HFrEF dataset, performance declined when codes were more numerous, overlapping, and multiassigned. These limitations do not reduce the value of our findings, but rather, where careful adaptation is warranted and form the basis for ongoing efforts to extend and improve this approach in additional IS evaluations of contextual determinants.

Looking ahead, this work will prioritize strengthening the end-to-end pipeline outlined in the tooling and operationalization framework, tightening data structuring and ingestion, standardizing reproducible reporting, and formalizing versioning and documentation to ensure auditable, integrable outputs. In parallel, a Delphi study with domain experts will systematically evaluate usability and interpretability, test alignment with theoretical constructs, and build consensus on codebook definitions, multicoding rules, and decision thresholds. Together, these efforts are designed to consolidate methodological rigor and elevate the practical use of AI-assisted qualitative analysis within IS.

## Appendix

Multimedia Appendix 1 Normalized Confusion Matrix — DistilBERT (LUS Dataset).

Multimedia Appendix 2 Normalized Confusion Matrix — DistilBERT (HRrEF Dataset).

## Abbreviations

AI: artificial intelligence
AI-RQA: AI-assisted rapid qualitative analysis for implementation science
AUPR: area under the precision-recall curve
BERT: bidirectional encoder representations from transformers
DistilBERT: distilled bidirectional encoder representations from transformers
ELECTRA: efficiently learning an encoder that classifies token replacements accurately
HFrEF: heart failure and reduced ejection fraction
IS: implementation science
LLM: large language model
LUS: lung ultrasound
ML: machine learning
NLP: natural language processing
PRISM: Practical, Robust Implementation and Sustainability Model
SLM: small language model
