# Supplementary figures and images for: AI-Assisted Rapid Quality Analysis in Implementation Science: Methodological Study
Source: JMIR AI. 2026 Apr 6;5:e81149. doi: 10.2196/81149 (PMC13096769; doi:10.2196/81149)

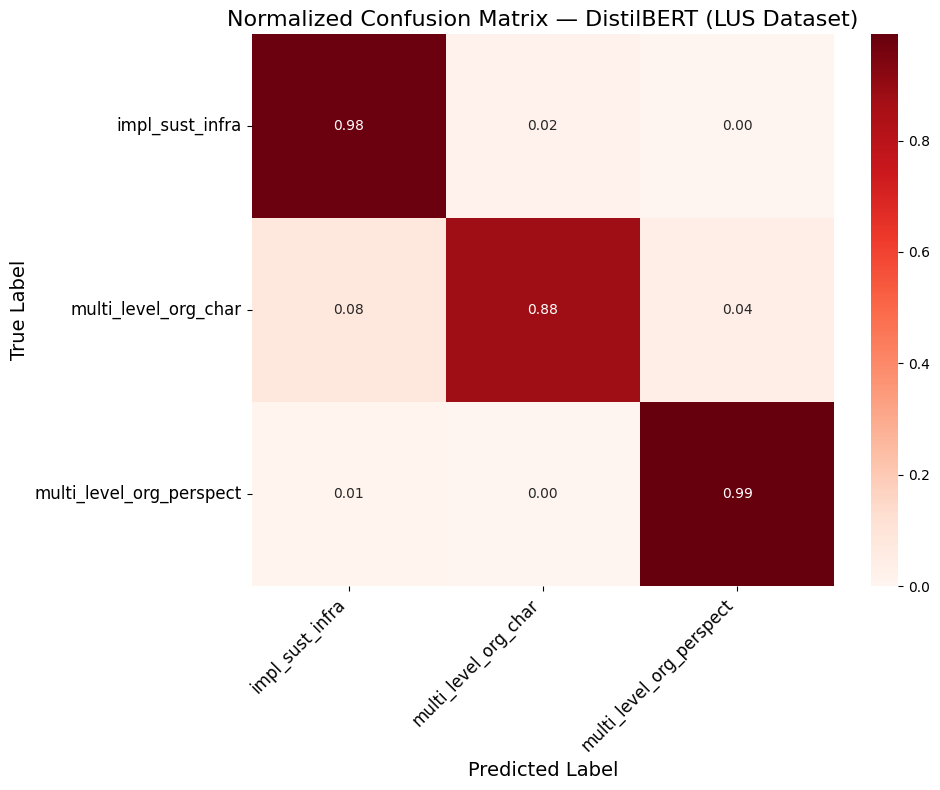

Supplement: Multimedia Appendix 1 [file ai_v5i1e81149_app1.png]

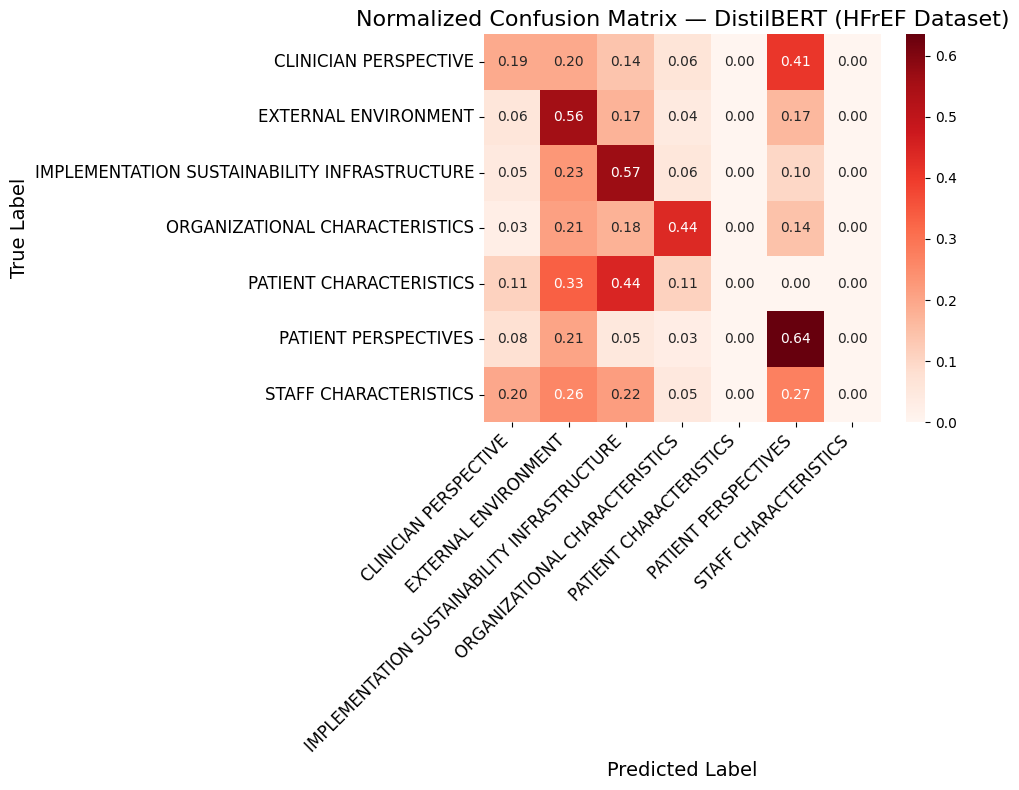

Supplement: Multimedia Appendix 2 [file ai_v5i1e81149_app2.png]
